# The effect of dexmedetomidine on the postoperative recovery of patients with severe traumatic brain injury undergoing craniotomy treatment: a retrospective study

**DOI:** 10.1186/s40001-024-01861-0

**Published:** 2024-04-30

**Authors:** Zhu Deng, Yong Gu, Le Luo, Lin Deng, Yingwei Li, Wanyong Huang

**Affiliations:** 1 Department of Neurosurgery, People’s Hospital of Guanghan City, No.9, Section3, Xi’an Road, Guanghan, Sichuan People’s Republic of China; 2 Department of Intensive Care Unit, People’s Hospital of Guanghan City, No.9, Section3, Xi’an Road, Guanghan, Sichuan People’s Republic of China

**Keywords:** Traumatic brain injury, Craniotomy, Dexmedetomidine, Delirium

## Abstract

**Background:**

Traumatic brain injury (TBI) has been a worldwide problem for neurosurgeons. Patients with severe TBI may undergo craniotomy. These patients often require sedation after craniotomy. Dexmedetomidine (DEX) has been used in patients receiving anesthesia and in intensive care units. Not much is known about the postoperative effect of DEX in patients with severe TBIs undergoing craniotomy. The purpose of this study was to explore the effects of postoperative DEX administration on severe TBI patients who underwent craniotomy.

**Methods:**

Patients who underwent craniectomy for severe TBI at our hospital between January 2019 and February 2022 were included in this study. The patients were admitted to the intensive care unit (ICU) after surgery to receive sedative medication. The patients were then divided into DEX and control groups. We analyzed the sedation, hemodynamics, and other conditions of the patients (hypoxemia, duration of ventilation during endotracheal intubation, whether tracheotomy was performed, and the duration in the ICU) during their ICU stay. Other conditions, such as delirium after the patients were transferred to the general ward, were also analyzed.

**Results:**

A total of 122 patients were included in this study. Among them, 53 patients received DEX, and the remaining 69 did not. The incidence of delirium in the general ward in the DEX group was significantly lower than that in the control group (*P* < 0.05). The incidence of bradycardia in the control group was significantly lower than that in the DEX group (*P* < 0.05). Other data from the DEX group and the control group (hypotension, hypoxemia, etc.) were not significantly different (*P* > 0.05).

**Conclusion:**

The use of DEX in the ICU can effectively reduce the incidence of delirium in patients who return to the general ward after craniotomy. DEX had no adverse effect on the prognosis of patients other than causing bradycardia.

## Background

Traumatic brain injury (TBI) is a serious traumatic disease that is a difficult to treat and is considered problem worldwide (especially for critically ill TBI patients) [1]. Approximately 0.5% of people experience TBI each year due to trauma [2]. TBI has always been difficult for neurosurgeons due to its high disability and mortality rates. Moreover, TBI not only causes disaster in patients themselves, but also places a heavy burden on families and society [2, 3]. According to the mechanism of injury, TBIs can be divided into two categories: primary injury (mainly including epidural hematoma, subdural hematoma, brain tissue contusion and axonal injury) and secondary injury (mainly including brain tissue edema and increased intracranial pressure) [4, 5]. According to the severity of the disease, TBI can be divided into mild (score of 13–15), moderate (score of 9–12), and severe (score ≤ 8) according to the Glasgow Coma Scale (GCS) [6–8].

For patients with TBI, a primary injury such as a subdural hemorrhage results in a series of secondary injuries. The inflammatory response of the nervous system, oxidative stress injury and nerve cell apoptosis are the main causes of these secondary injuries [9, 10]. Among these changes, the inflammatory response of the nervous system, caused by trauma, is thought to be the key factor that triggers a series of reactions. Its main response changes to glial activation, peripheral inflammatory cell infiltration and inflammatory mediator release [9]. For TBI patients, an appropriate inflammatory response plays a positive role in damage repair. However, severe TBI often causes this inflammatory response to be excessive and persistent, resulting in pathophysiological changes that are difficult to control. The series of uncontrollable pathophysiological changes induced by TBI is ultimately responsible for its high disability and mortality rates [2, 3, 9]. Therefore, neurosurgeons have always hoped to reduce the harm caused by TBIs by controlling the excessive inflammatory response to improve neurological function.

Dexmedetomidine (DEX), a highly selective α-2 adrenergic receptor agonist, has been used in patients receiving anesthesia and in intensive care units (ICUs) [11]. DEX has good performance in terms of preventing anxiety, providing analgesia and providing sedation to patients [11]. A study of TBI patients revealed that DEX can inhibit an excessive inflammatory response by inhibiting the activation of some inflammasomes, thereby exerting a certain protective effect on the nervous system of TBI patients in the early stage [12]. In fact, DEX has been used in the management of patients admitted to the ICU for severe TBI. DEX can alleviate agitation, prevent the occurrence of paroxysmal sympathetic hyperactivity, and reduce the occurrence of delirium in patients with severe TBI in the ICU. However, DEX has side effects, such as hemodynamic changes [13–15].

Although DEX has a clear effect on patients with severe TBI, little is known about the postoperative effect of DEX in patients with severe TBI who underwent craniotomy. Therefore, the purpose of this study was to determine whether DEX is equally safe and effective for treating severe TBI after craniotomy and whether DEX will have a long-term impact on patient prognosis.

## Methods

### Study subjects

Retrospectively and consecutively, the subjects of this study were all severe TBI patients who underwent craniotomy in the Department of Neurosurgery, People's Hospital of Guanghan City, from January 2019 to February 2022. Patients who were older than 80 years and younger than 18 years, followed up for less than half a year, lacked clinical data, died, had a second surgery, or did not receive sedation and patients or family members who declined to participate in this study were excluded. All patients underwent cranial CT on the first day, the third day and 1 week after craniotomy. Patients who experienced postoperative rebleeding or incomplete hematoma removal were excluded (postoperative intracranial hematoma > 5 ml). We also evaluated other relevant organ systems in patients with delirium and agitation. Patients were excluded if metabolic abnormalities and other complications were present. All family members of the patients signed informed consent. The article follows the STROBE statement and was approved by the People's Hospital of Guanghan City Ethics Committee.

### Data collection and outcome assessment

The clinical characteristics and demographic data were recorded by the authors of the study after the patients were admitted. The data included sex, age, preoperative GCS score, and preoperative pupil condition (mydriasis). After surgery, patients were treated with sedative drugs in the ICU (dexamethasone (DEX) or not), degree of sedation (Richmond Agitation-Sedation Scores (RASS); patients were then transferred to the ICU immediately after surgery, and scoring began upon admission to the ICU. Hemodynamics during sedation included: heart rate and arterial blood pressure. Blood oxygen saturation during sedation, the duration of sedation, the ventilation time for endotracheal intubation were also recorded. It is unclear whether tracheotomy is acceptable. The duration in the ICU. When vital signs were stable (no vasopressors are needed to maintain blood pressure) and blood oxygen saturation was greater than 90% under ordinary oxygen inhalation (no ventilator is needed to assist breathing), the patient was transferred to the general ward. Delirium was confirmed after the patient was transferred to the general ward. The total length of hospital stay. Condition at discharge: GCS score at discharge. Long-term outcomes were assessed using the Glasgow Outcome Scale (GOS) (Table 1). In addition, if the patient's heart rate was lower than 55, it was considered to indicate bradycardia; if the systolic blood pressure was less than 90, it was considered to indicate hypotension; if the blood oxygen saturation was lower than 90, it was considered to indicate hypoxemia. A patient with a RASS score greater than or equal to 2 during sedation was considered to have a poor sedation effect. We assessed patients for delirium risk by using the validation of the delirium rating scale-revised-98 (DRS-R-98). A score of ≥ 1 for the 3 diagnostic items indicated the presence of delirium in a patient. Disturbances due to delirium and agitation due to metabolic conditions were avoided.

**Table 1:** Glasgow Outcome Scale (Glasgow Outcome Scale, GOS)

Score	Description
1	Death
2	Vegetative state
3	Severe disability
4	Moderate disability
5	Good recovery

### Therapy method

All the patients underwent craniotomy at the People's Hospital of Guanghan City by highly experienced and well-trained neurosurgeons. The patients were admitted to the ICU after surgery to receive sedative medication. The sedative drug in the control group was propofol plus midazolam, while the sedative drug in the experimental group was DEX in combination with propofol. In terms of analgesia, our drug of choice is fentanyl. A RASS score ≥ 2 indicated that the sedative effect was poor, and the sedation regimen needed to be adjusted (appropriately increasing the dose of sedative drugs). All patients received essentially the same treatment regimen, with the exception of treatment differences on appeal.

### Statistical analyses

All the data we collected were analyzed by R version 4.2.2. All data analyses were performed only between the experimental group (DEX group) and the control group. Continuous variables are expressed as the mean ± standard deviation (for example, age, preoperative GCS score, ventilation time at endotracheal intubation, total length of hospital stay, etc.). We used the numbers and percentages to represent the categorical variables (for example, sex, mydriasis, bradycardia, and delirium after transfer to the general ward). We used the Kolmogorov‒Smirnov test for the normality of continuous variables, and the t test was subsequently used to compare the experimental group and the control group. Additionally, we used Pearson’s χ^2^ test or Fisher’s exact test for comparisons between categorical variables. A difference was considered to be statistically significant when P was ≤ 0.05.

## Results

### Clinical characteristics

A total of 157 patients received craniotomy at People's Hospital of Guanghan City from January 2019 to February 2022. Among those patients, 23 died after surgery, 5 patients were transferred to a high-level hospital for continued treatment, 3 patients abandoned treatment after surgery, and 4 patients were lost to follow-up after discharge. After excluding these patients, we eventually enrolled 122 patients in this study. Of the patients included in the study, 53 received DEX, and the remaining 69 did not.

The demographic and clinical data of the patients are shown in Table 2. The results showed that there were no significant differences in sex, age, preoperative GCS score, or preoperative pupil condition (mydriasis) between the DEX group and the control group (*P* > 0.05).

**Table 2:** Demographic and clinical characteristics data of the dexmedetomidine group and the control group

Variable	Dexmedetomidine	Control	*P* value
Gender			0.8212^a^
Male	41	51	
Female	12	18	
Age (years)			0.8357^b^
Mean ± SD	60 ± 13	60 ± 12	
Preoperative GCS			0.1183^b^
Mean ± SD	8 ± 3	9 ± 2	
The preoperative			0.08191^a^
Pupil condition	Yes	10 24	
(Mydriasis)	No	43	*45*

^a^*P* value: Pearson's *χ*^2^ test or Fisher's exact test; ^b^*P* value: *t* test

### ICU data

Sedation with DEX was initiated as soon as the patient was admitted to the ICU, and the duration of use was 44 ± 40 h. The mean rate of administration was 0.53 ± 0.25 μg/kg/h, the mean minimum rate was 0.26 ± 0.11 μg/kg/h, and the mean maximum rate was 0.98 ± 0.21 μg/kg/h. During IUC, 27 patients in the DEX group developed bradycardia. A total of 19 people in the control group developed bradycardia. There was a significant difference between the two groups (*P* < 0.05). Other data from the DEX group and the control group collected during the ICU stay (hypotension, hypoxemia, RASS score, ventilation time of endotracheal intubation, whether tracheotomy was performed, and duration in the ICU) were not significantly different (*P* > 0.05).

### General ward and other data

After the patients were transferred to the general ward, a total of 6 patients in the DEX group developed delirium, and a total of 19 patients in the control group developed delirium. There was a significant difference between the two groups (*P* < 0.05). Moreover, the total length of hospital stay, GCS score at discharge and GOS score six months after discharge did not significantly differ between the DEX group and the control group (*P* > 0.05). Our subgroup analysis of patients who presented with delirium revealed no significant difference in the preoperative (Tables 3 and 4) GCS score or postoperative cranial CT (*P* > 0.05).

**Table 3:** Shows the ICU data of the dexmedetomidine group and the control group

Variable	Dexmedetomidine	Control	*P* value
RASS score ≥ 2			0.5113^a^
Yes	3	50	
No	62	7	
Bradycardia			0.01406^a^
Yes	26	19	
No	27	50	
Hypotension			0.09531^a^
Yes	29	20	
No	24	49	
Hypoxemia			1^a^
Yes	5	6	
No	48	63	
The duration of sedation (hours)	44 ± 40	56 + 46	0.1347^b^
Ventilation time of endotracheal intubation	39 ± 26	44 ± 33	0.3401^b^
Tracheotomy			0.945^a^
Yes	25	44	
No	18	35	
The duration of ICU (days)	5.3 ± 5.1	5.3 ± 4.6	0.961^b^

^a^*P* value: Pearson's *χ*^2^ test or Fisher's exact test; ^b^*P* value: *t* test

**Table 4:** General ward and other data of the dexmedetomidine group and control group

Variable	Dexmedetomidine	Control	*P* value
Delirium after transfer back to the general ward			0.04847^a^
Yes	6	19	
No	47	50	
The total length of hospital stay (days)	36.6 ± 28.6	30.7 ± 12.7	0.1315^b^
GCS score at discharge	12 ± 3	11 ± 2	0.6562^b^
GOS score six months after surgery	3.3 ± 1.3	3.5 ± 1.0	0.3041^b^

^a^*P* value: Pearson's *χ*^2^ test or Fisher's exact test; ^b^*P* value: *t* test

## Discussion

We conducted a retrospective analysis of 122 patients who underwent craniotomy in the Department of Neurosurgery of our hospital and found that the incidence of delirium in patients who returned to the general ward in the DEX group was significantly lower than that in the control group, but the manifestation of delirium significantly increased the incidence of bradycardia. In addition, DEX did not increase the occurrence of other adverse events. There was no significant difference in length of ICU stay or total length of stay between the two groups. In terms of patient recovery, DEX did not adversely affect patient prognosis.

Many patients with severe TBI are transferred to the ICU for more effective treatment, especially for those with severe TBI who have undergone craniotomy [16]. Because of the severe nature of TBI itself, the need for craniotomy and other reasons, these patients often exhibit agitation [3, 17]. Once a patient has this condition, it usually needs to be controlled with medication. In some cases, restraint bands may even be used to protect patients [18]. Propofol, midazolam, and other agents are often used to sedate agitated patients during ICU stays [19]. A descriptive study by Bilodeau et al. revealed a favorable sedative effect of DEX in patients with TBI [13]. For severe TBI patients undergoing craniotomy, these sedatives can also have a significant effect [2]. DEX, a new highly selective α-2 adrenergic agonist, has been approved by anesthesiologists and ICU physicians. A small number of studies have shown satisfactory results for sedation with DEX in severe TBI patients who had not undergone craniotomy during an ICU stay [20, 21]. As an increasing amount of research has been conducted on DEX in TBI patients, the benefits of DEX on TBI patients have gradually been discovered. A study by Hao et al. revealed DEX to be more effective than propofol at controlling the overstress response after TBI [22]. In addition, DEX can reduce intestinal tissue damage by improving the inflammatory response after TBI [23]. The protection of brain function is important for patients with TBI, and Wang et al. reported no adverse effects of DEX use on brain function [24]. DEX is also gradually being used in the sedation of patients with TBI. Our study revealed that DEX also had a significant sedative effect on severe TBI patients undergoing craniotomy.

Currently, adverse events related to DEX mainly manifest as a result of its effect on hemodynamics. DEX has been found to induce hypotension during the ICU stay [25]. This was also observed in our study, but DEX did not significantly affect hypotension incidence compared to that in the control group. However, our study revealed that the DEX treatment group had a significantly greater incidence of bradycardia than the control group. DEX has also been used in anesthesiology departments and ICUs in the recent years, but the mechanism of its influence on the hemodynamics of patients is still unclear. Currently, the mechanism of action of DEX, which is recognized by the academic community, is as follows: as an α-2 adrenergic agonist, DEX can activate presynaptic and postsynaptic α-2 receptors, thereby causing contraction of peripheral blood vessels, relaxation of peripheral blood vessels and reflex bradycardia, which can cause increased blood pressure or a decline in blood pressure. Moreover, DEX can significantly reduce the occurrence of stress reactions, which leads to a reduced likelihood of catecholamine secretion due to the stress response; thus, DEX does not cause a hemodynamic effect on catecholamine secretion [26, 27].

Delirium, an acute mental state change, is characterized by inattention, arousal disorders and mental disorders [28]. However, the presence of delirium caused irreversible damage to the brain function of patients, significantly increased the total length of hospital stay, significantly increased the cost of hospitalization, and even seriously affected the prognosis [28, 29]. Delirium is highly common in patients with severe TBI and can occur at any time during a patient's hospital stay, and studies suggest that up to 70% of patients present during a hospital stay [28, 30]. Surgery is also an important cause of delirium, and the incidence of delirium after emergency surgery is 20–45%. Delirium usually occurs in these patients 2–5 days after surgery, but it is possible throughout the hospital stay [31]. Because treating delirium in the general ward can be more difficult than treating it in the ICU, our study evaluated the occurrence of delirium in the general ward. Our subjects were patients with severe TBI who had also undergone craniotomy, both of which could have caused a high incidence of delirium during their hospital stay. Our study only measured the incidence of delirium in patients who were transferred back to the general ward after stabilizing. Some of these patients may have developed delirium during their stay in the ICU and may have been treated. Therefore, we do not have a particularly high incidence. In our study, we were surprised to find that the use of DEX during hospitalization in the ICU can effectively reduce the incidence of delirium in patients in general wards.

The main shortcomings in this study are as follows. First, this was a retrospective study, and the retrospective nature of the study itself may have introduced bias. Second, the sample size of this study was relatively small, so there were certain difficulties in performing statistical analysis for those events with a very low incidence (e.g., RASS score ≥ 2), which also leads to an increased risk of bias. We hope to conduct a larger sample size and more in-depth study in the future. In addition, the small sample size prevented us from further subdividing the types of TBI patients, which also resulted in our findings not clarifying whether DEX has the same effect on extradural hematoma and subdural hematoma. However, these findings also point to the next step in our research. Fourth, this study only preliminarily explored patients who underwent craniotomy. There was no further research on the various craniotomy methods used in this study. Finally, this study was aimed at TBI patients, and whether this approach is applicable for non-TBI patients who undergo craniotomy needs to be further studied. In addition, due to the absence of data, our study analyzed only the occurrence of delirium in the general ward and did not evaluate the occurrence of delirium during patients' ICU stay.

## Conclusion

In this study, the use of DEX in the ICU effectively reduced the incidence of delirium in patients who returned to the general ward after craniotomy. The sedative effect of DEX was satisfactory. In addition, DEX use in the ICU did not cause adverse events in patients other than an increased incidence of bradycardia.

## Data Availability

The data that support the findings of this study are available from the corresponding author upon reasonable request.

## Abbreviations

DEX: Dexmedetomidine
TBI: Traumatic brain injury
GCS: Glasgow Coma Scale
ICU: Intensive care units
RASS: Richmond Agitation-Sedation Scores
GOS: Glasgow Outcome Scale
DRS-R-98: Delirium rating scale-revised-98
